# Diurnal functional and anatomical changes in X-linked retinoschisis

**DOI:** 10.1007/s00417-023-06106-0

**Published:** 2023-06-09

**Authors:** Luca Mautone, Yevgeniya Atiskova, Vasyl Druchkiv, Martin Stephan Spitzer, Simon Dulz

**Affiliations:** Department of Ophthalmology, University Medical Center Hamburg-Eppendorf, Martinist. 52, 20246 Hamburg, Germany

**Keywords:** Retinoschisis, Optical coherence tomography, X-linked retinoschisis, Diurnal variation, RS1 gene mutation, Microperimetry

## Abstract

**Background:**

To investigate the changes in macular cystic schisis (MCS) and sensitivity during the day in X-linked retinoschisis (XLRS) patients.

**Methods:**

Treatment-naïve patients with genetically verified XLRS underwent best-correlated visual acuity (BCVA) testing with ETDRS charts, spectral domain optical coherence tomography, and microperimetry (MP) twice a day, at 9 a.m. and 4 p.m., to measure changes in central retinal thickness (CRT), macular volume (MV), average threshold (AT), and fixation stability parameters (P1 and P2).

**Results:**

At baseline, the BCVA of the 14 eyes of 8 patients amounted 0.73 (± 0.23) LogMAR. Between timepoints, the BCVA increased in 3.21 letters (*p* = .021), the AV improved in 1.84 dB (*p* = .03, 9.73%), the CRT decreased in 24.43 µm (*p* = .007, − 4.05%), and the MV dropped in 0.27 µm^3^ (*p* = .016, − 2.68%). P1 and P2 did not variate. The collapse of the MCS led to the reduction of macula thickness. CRT at baseline correlated with the decrease of CRT (Spearman’s *ρ*: − 0.83 [*p* = .001]). Age and change of BCVA, CRT, and AV did not correlate among one another. Eyes with disrupted ellipsoid zone showed a more prominent change in CRT (*p* = .050). Photoreceptor outer segment length and integrity of the external limiting membrane and cone outer segment tips were not associated with BCVA, AT, or CRT variation.

**Conclusion:**

Eyes of treatment-naïve XLRS patients show diurnal macular thickness and function changes. Eyes with pronounced macular thickness show a greater reduction of the MCS. These results should be taken into consideration in upcoming clinical trials in XLRS.

**Trial registration number:**

Institutional Review Board of the Hamburg Medical Chamber (*Ethik-Kommission der Ärztekammer Hamburg*): 2020–10,328.

**Supplementary Information:**

The online version contains supplementary material available at 10.1007/s00417-023-06106-0.


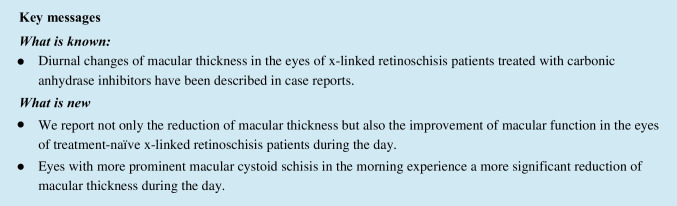


## Introduction

X-linked retinoschisis (XLRS, OMIM 312,700) is a monogenetic hereditary retinal dystrophy with an estimated prevalence between 1 in 15,000 and 1 in 30,000 caused by mutations of the retinoschisis-1 gene (RS1)[1]. The RS1 gene codes for retinoschisin (RS), a 224 amino acid extracellular expressed protein that plays a pivotal role in the cellular adhesion and maintenance of retinal structure [2]. Genetic mutation of the RS1 gene drives to a retinal phenotype characterized by distinctive features such as peripheral retinoschisis and the distinctive macular cystic schisis (MCS).

Similar to the well-described diurnal variations of macular thickness in various retinal diseases with cystoid macular edema [3–6], two case reports showed the progressive reduction of macular thickness caused by the diminution of MCS during the day in XLRS patients treated with carbohydrase inhibitors (CAI) and that underwent light exposition during the sleep [7, 8].

To date, there is no approved therapy for this disease. The role of topical and systemic CAI remains an object of discussion due to the lack of prospective randomized clinical trials and diverging results of studies. Several investigations reported a reduction of MCS accompanied by a BCVA increase under CAI therapy [9–11], whereas others described only an improvement of MCS without significant changes in visual acuity [12]. Trials to investigate potential gene therapies in XLRS were conducted. Despite the preclinical achievements [13–16], both phase I/II trials for ocular RS1 adeno-associated virus gene augmentation therapy in humans did not report any significant MCS reduction or retinal function improvement [17, 18].

To date, it has not been investigated whether diurnal fluctuation of the volume of the MCS is part of the natural course of the disease and may occur independently from therapeutic interventions. Furthermore, the diurnal variation of macular function in XLRS eyes has yet to be the object of research. This study examined diurnal morphological and functional changes of the macula treatment-naïve patients with genetically confirmed XLRS. This investigation aimed to evaluate if naturally occurring architectural and functional fluctuations in XLRS should be considered in future interventional clinical trials.

## Materials and methods

### Study protocol

This cross-sectional, observational, single-center cohort study was conducted at the Ophthalmology Department of the University Medical Center Hamburg-Eppendorf, Germany. This study adhered to the tenets of the Declaration of Helsinki, and prior approbation was obtained from the Institutional Review Board of the Hamburg Medical Chamber (*Ärztekammer* 2020–10,328). Written informed consent was obtained from each adult subject (aged 18 years or older) and the parents of minor subjects, with assent from the minors.

Males at least of an age of 7 years with confirmed RS1 gene mutation were included. The exclusion criteria were ongoing therapy with CAI, intraocular surgery within 3 years before enrollment, and history of other ocular diseases.

Best-correlated visual acuity (BCVA) measurement using the Early Treatment Diabetic Retinopathy Study (ETDRS) chart, spectral domain optical coherence tomography (OCT), and microperimetry (MP) were performed twice, at 9 a.m. and 4 p.m., respectively. Furthermore, every patient underwent a complete ophthalmological examination, including Goldmann applanation tonometry, slit-lamp biomicroscopy, and fundus examination. Indirect fundoscopy was performed under miotic conditions to avoid influencing BCVA measurement, OCT, and MP.

### OCT analysis

Horizontal cross-sectional OCT images within the fovea’s horizontal and vertical 20° were generated using Spectralis OCT 2 (Heidelberg Engineering, Heidelberg, Germany). Centration of scans was reviewed and corrected manually if not appropriately centered on the macula. Automated eye alignment eye-tracking software and AutoRescan feature were used to automatically place 4 p.m. follow-up scans in the exact location as the baseline scan. To measure macular thickness and volume, the ETDRS-type grid was positioned on the center of the fovea. Retinal thickness (CRT) and macular volume were automatically assessed with the Heidelberg Eye Explorer (version 3.1.4; Heidelberg Engineering, Heidelberg, Germany). Two observers (LM and SD) independently determined the presence of retinal defects and MCS. Specific OCT findings were evaluated in a radius area 1 mm from the foveal center, representing the anatomical location of the macula, as previously described [19]. External limiting membrane (ELM), cone outer segment tips (COST), and ellipsoid zone (EZ) were also assessed according to the literature’s definitions to examine outer retinal photoreceptors’ integrity in the macula [20, 21]. The outer photoreceptor segment (PROS) length, assessed at the foveal center as a perpendicular distance from the EZ’s posterior surface to the retinal pigment epithelium’s anterior surface, was measured manually [19].

### Macular sensitivity assessment

Macular sensitivity was assessed with fundus-controlled MP (MAIA, Macular Integrity Assessment, Centervue, Padova, Italy). The average retinal sensitivity of the test grid (average threshold) and fixation stability parameters P1 and P2 were recorded using the large 10–2 grid patterns (68 test loci) covering a 20° field centered on the fovea. Goldman III (0.43° in diameter) white stimuli with 200-ms duration were presented with a 4–2 testing strategy. The follow-up function adopted the baseline reference test to ensure registration of the same test loci for the testing at 4 p.m.

### Statistical analysis

A mixed regression model, paired *T*-test, Wilcoxon signed rank, independent *T*-test, Mann–Whitney test, and Spearman-Rank correlation were applied for statistical analysis with R Core Team [22]. Significant levels were set at *p* < 0.05. More details about the performed statistical methods are described in the supplementary information.

## Results

The clinical data of the enrolled 14 eyes of 8 patients are summarized in Table 1. Two eyes of 2 subjects were excluded due to the atrophic macula and missing MCS. Patient 2 interrupted the MP for the right eye due to discomfort, and therefore, this examination was not considered for analysis. The mean age was 26.3 (± 6.67) years and did not significantly correlate with CRT, read letters, average threshold, and fixation stability P1 and P2 measured at 9 a.m. and PROS length (Table S1).

**Table 1:** Clinical characteristics and demographics. OR, right eye; OS, left eye; CP, cryopexy; PPV, pars plana vitrectomy. Patients marked with * and † are siblings

Patient	Age (years)	Eye	History of intraocular surgery	Genotype
1	26	OR OS		c.(184 + 1_185-1)_(552 + 1_523-1)del
2	31	OR OS	CP CP, PPV	Genetically confirmed but not further specified
3*	31	OR OS		c.589C > T
4*	25	OR OS		c.589C > T
5	38	OS		c.325G > C
6^†^	26	OS		c.276G > C
7^†^	17	OR OS		c.276G > C
8	17	OR OS	PPV, cataract surgery	c.626G > A

The mean BCVA was 0.73 (± 0.23) LogMAR at 9 a.m. and 0.74 (± 0.33) LogMAR at 4 p.m. (mean change: 0.01 [± 0.32] LogMAR, *p* = 0.869). At baseline, CRT did not significantly correlate to BCVA and MP parameters (Table S1). The read letters at 9 a.m. correlated significantly with the average threshold (Spearman’s *ρ*: 0.63 [*p* = 0.022]) at baseline and with the PROS length (Spearman’s *ρ*: 0.69 [*p* = 0.006]). Furthermore, the PROS length also significantly correlated to the average threshold at baseline (Spearman’s *ρ*: 0.69 [*p* = 0.009]). The average threshold at the morning control correlated to fixation stability P2 at 9 a.m. (Spearman’s *ρ*: 0.48 [*p* = 0.018]), which, in turn, correlated to fixation stability P1 (Spearman’s *ρ*: 0.95 [*p* = 0.0001]).

**Fig. 1:**
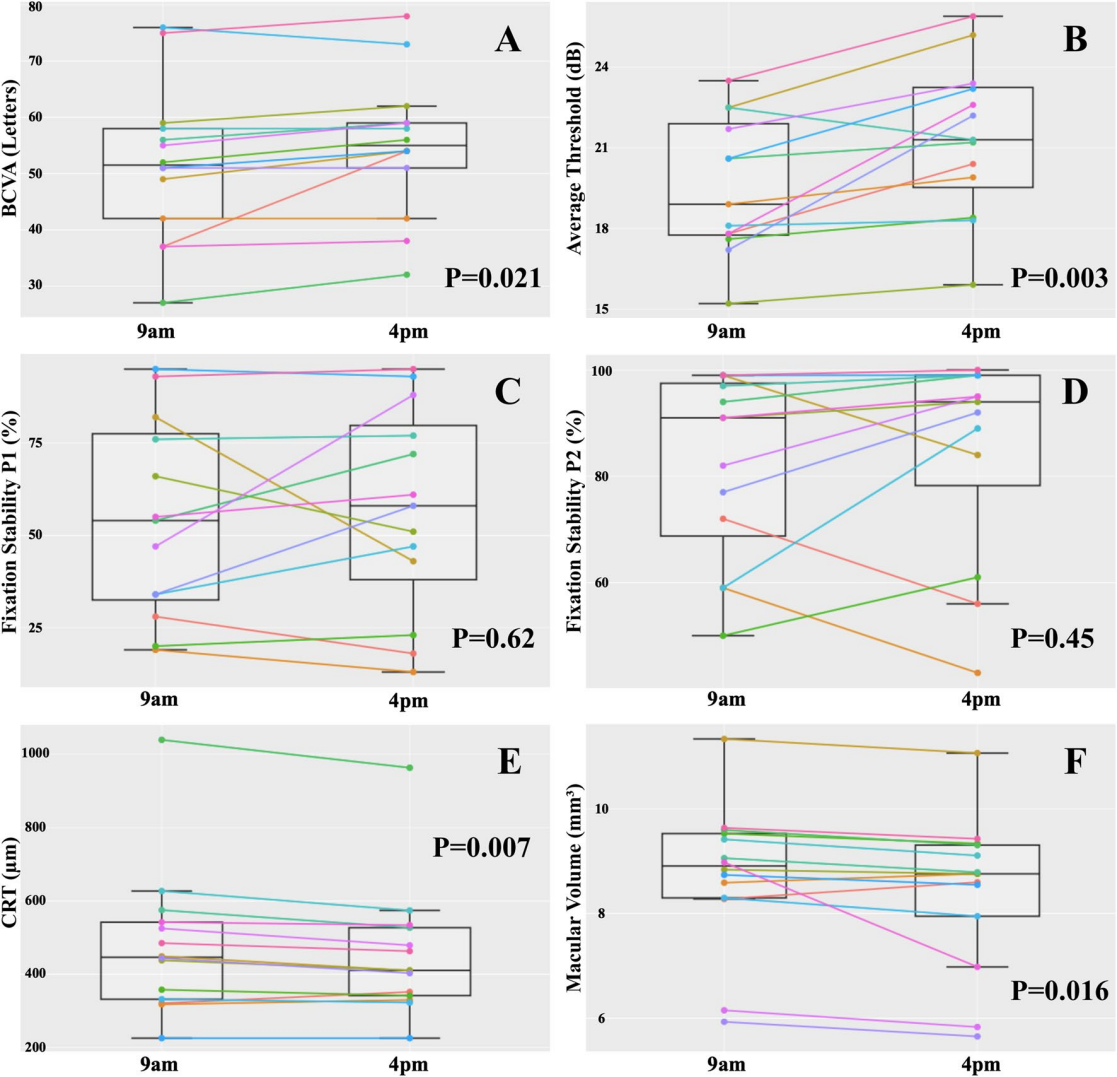
**A** Change of the best-correlated visual acuity (BCVA) measured by read letters (*y*-axis). **B** Change of the average threshold (dB, *y*-axis). **C** Change of the fixation stability P1 (%, *y*-axis). **D** Change of the fixation stability P2 (%, *y*-axis). **E** Change of the central retinal thickness (CRT, µm, *y*-axis). **F** Change of the macular volume (µm.^3^, *y*-axis)

The diurnal changes in macular anatomical and functional parameters are illustrated in Fig. 1. The read letters increased from 51.79 (± 13.59) at baseline to 55.00 (± 12.25) in the afternoon examination (mean change: 3.21 [± 4.58] letters, *p* = 0.021). An improvement of the average threshold was observed from 19.70 (± 2.57) dB at 9 a.m. to 21.62 (± 2.81) dB at 4 p.m. (mean change: 1.84 [± 1.77] dB, *p* = 0.003) and its mean relative change was 9.73 (± 9.72) % (*p* = 0.003). Fixation parameters P1 and P2 did not significantly vary between timepoints: P1 was 56.92 (± 25.7) % at 9 a.m. and 59.67 (± 27.05) % at 4 p.m. (mean change: 2.77 [± 19.55] %, *p* = 0.62), while a P2 of 84.92 (± 15.01) % at 9 a.m. and 87.08 (± 18.37) % at 4 p.m. was measured (mean change: 2.85 [± 13.23] % *p* = 0.45).

**Fig. 2:**
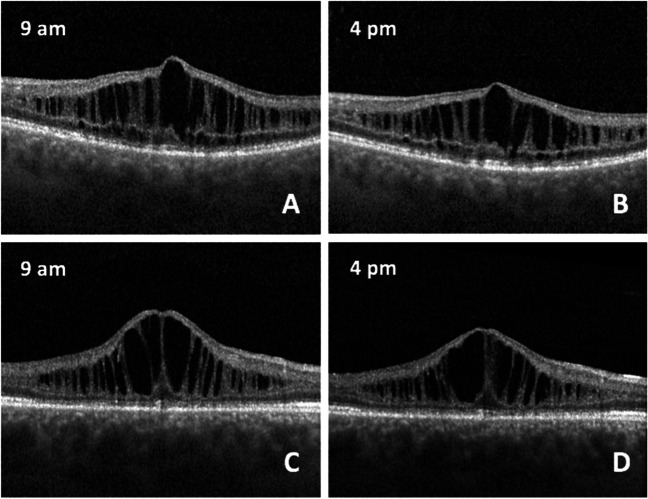
Patient #7, spectral-domain OCT (SD-OCT) of the left eye (**A** and **B**): central retinal thickness (CRT) 525 µm and macular volume (MV) 6.15 µm^3^ at 9 a.m. (**A**). CRT 479 µm and MV 5.83 µm^3^ at 4 p.m. (**B**). Patient #4, SD-OCT of the right eye (**C** and **D**). Central CRT 575 µm and MV 9.06 µm^3^ at 9 a.m. (**C**). CRT 527 µm and MV 8.79 µm.^3^ at 4 p.m. (**D**)

A significant decrease of CRT from a median of 446.50 (338.50; 537.75) µm at baseline to 410.50 (344.50; 515.00) µm at the afternoon control was shown (mean change: − 24.43 [± 28.52] µm, *p* = 0.007). The mean relative change of CRT was − 4.05 (± 5.53) % (*p* = 0.013). The collapse of MCS led to the reduction of macular thickness, as demonstrated in Fig. 2. The median change of the macular volume from 8.91 (8.37; 9.50) µm^3^ to 8.76 (8.10;9.26) µm^3^ between the intraday controls was − 0.27 (− 0.31; − 0.19) µm^3^ and also statistically significant (*p* = 0.016). The median relative change of the macular volume amounted to − 2.68 (− 3.99; − 2.04) % (*p* = 0.009). Age was associated with the variations of CRT, BCVA, and average threshold (Table S1).

CRT at baseline correlated with decreased of CRT between timepoints (Spearman’s *ρ*: − 0.83 [*p* = 0.001]). Eyes with disrupted EZ showed a higher diurnal variation of CRT compared to eyes with intact EZ (− 41.00 [± 22.73] µm vs − 12.00 [± 27.00] µm, respectively; *p* = 0.050). The diurnal CRT variation did not significantly correlate to PROS length or ELM and COST’s integrity (Table S2–S4). No correlation between the diurnal change of CRT, BCVA, and the average threshold was observed (Table S1). In addition, the diurnal variation of BCVA and average threshold did not correlate to any assessed morphological and functional parameters (Table S1–S4).

## Discussion

The MCS represents a hallmark feature of the XLRS. Beyond the improvement of visual function, the reduction of MCS is an important endpoint of the treatment with CAI and current and upcoming clinical trials. The presented case series provides evidence of diurnal improvement of the macular function and reduction of macular thickness due to the collapse of MCS in treatment-naïve XLRS patients.

In this study, the reduction of macular thickness, objectified as a decrease of the CRT and macular volume, and the improvement of macular function, assessed by MP and BCVA testing, were described between 9 a.m. and 4 p.m. As shown here, the diurnal fluctuation of parameters might confound measurements. Hence, the available data about the off-label efficacy of CAI [9–12] and gene therapies [17, 18] could be susceptible to bias. These circadian changes should be regarded for the follow-up controls and similarly for the design of future clinical trials. Therefore, OCT, BCVA testing, and MP should be performed at the same timepoint.

Previously, fluctuations of the macular thickness in XLRS patients at 6-month intervals were described [23]. However, the first evidence of diurnal reduction of macular thickness was reported in two small case series [7, 8]. In this study, a mean CRT reduction of 4.05% was measured. The described decrease of the macular thickness due to the collapse of MCS is lower compared to both above-mentioned publications (ca. 25%); yet, several differences in the study designs can explain this discrepancy. Contrary to Abalem et al. and Rubinstein et al., patients treated with CAI were excluded, eyes with signs of macular atrophy in OCT were included, patients were not actively exposed to particular light conditions during the night, and a different amount of controls were performed different timepoints [7, 8].

Many mechanisms could underline the circadian macular function and thickness changes in XLRS. RS regulates the fluid balance between intra- and extracellular space through binding to the Na/K-ATPase in photoreceptors and bipolar cells [2], and its expression and secretion underlie a circadian rhythm reaching its peak at night [24–26]. This circadian mechanism could explain the diurnal macular recovery due to the higher expression of RS. In addition, the Na/K-ATPase itself underlies its own circadian activity with its apex during the day [7]. Assuming that an RS1 gene mutation carries a partial to complete loss of function of RS, the raised ATPase activity could also be responsible for the diurnal reduction of the macular thickness. Another hypothesis for the diurnal changes refers to the interaction of RS with the membrane L-type voltage-gated calcium channels (VGCCs), which are essential in synaptic transmission and overall calcium homeostasis [24, 25]. The higher nocturnal expression level of RS causes a decrease in VGCCs, potentially explaining the greater MCS and reduced macular function in the morning.

The diurnal improvement of visual acuity and macular sensitivity were observed, but as a psychophysical parameter, a learning effect cannot be excluded. To our knowledge, no data about intradiurnal test–retest variability of MP-parameters and BCVA for XLRS patients has been published in the literature. Indeed, several studies investigating test–retest variability of MP in patients with and without pathologic macular findings outlined increased sensitivity between the first and the second test, but not in subsequent tests [27, 28]. The mean improvement of the average threshold in this study was 1.84 dB, which differs from the one indicated in the studies mentioned above (± 3.81 dB and ± 4.3 dB). Test–retest intervisit variability of functional parameters for XLRS patients was previously investigated adopting controls every sixth month [23]. The proposed threshold for significant change in macular sensitivity assessed by MP is not applicable to our results due to the different MP device and test strategy. Furthermore, the here-described diurnal BCVA improvement of 3.21 letters approaches the previously described threshold for a statistically significant change of BCVA (3 and 7 letters in the worst and better eye, respectively) [23]. However, the estimated fluctuations of anatomical and functional parameters between visits could also have been influenced by the here reported diurnal changes.

The diurnal variation of CRT correlated to the CRT at baseline. Therefore, eyes with more pronounced MCS tend to experience a greater reduction of the MCS. The integrity of the macular layers might partially explain this result since an association between EZ and CRT variation was found. Indeed, the disruption of EZ is correlated to a lower visual acuity [29], and it might signalize the structural damage, which correlates to severer dysregulation of the mechanisms connected to the RS protein. No other association with other macular morphological parameters was identified.

The reduction of CRT did not correlate with the change in visual acuity or macular sensitivity assessed by MP. This finding and the association of the PROS length with the BCVA and the macular sensitivity at baseline also support the hypothesis that the macular function primary depends on the integrity of macular architecture rather than MCS extent [19, 29–31]. However, the BCVA and average threshold significantly improved in the afternoon control. As the intraretinal cystoid spaces of the MCS increase the cone receptor spacing in the fovea leading to poor visual function [32], the decrease of MCS during the day could mechanically reduce the distance between cone receptors and contribute to the rise of function.

This study has several limitations beyond the small number of subjects and included related patients. Although most of the enrolled patients underwent a MP examination before this study (data not shown), no microperimetry and BCVA measurements were performed as training before the functional test of this study. Furthermore, the function and macular thickness variation was sampled only at two timepoints and not throughout the day. In conclusion, treatment-naïve XLRS patients experience diurnal changes in macular thickness and function with improved macular sensitivity (BCVA and average threshold assessed by MP) and reduced macular thickness (CRT and macular volume). Therefore, we advocate a time-adapted protocol in patients with MCS in XLRS, as visual function and retinal morphology measurements are essential endpoints in clinical trials. The diurnal decrease of CRT correlated only with the CRT at baseline and the disruption of the EZ. Notably, the diurnal changes of functional parameters and MCS were not associated with the age or among each other. The PROS length and the integrity of COST and ELM did also not influence the circadian changes of macular function and thickness. Further investigations with a higher number of participants are needed to confirm these results.

### Supplementary Information

Below is the link to the electronic supplementary material.

Supplementary file1 (DOCX 31.1 KB)
