# Supplementary material for: Diurnal functional and anatomical changes in X-linked retinoschisis
Source: Graefes Arch Clin Exp Ophthalmol. 2023 Jun 9;261(11):3307–13. doi: 10.1007/s00417-023-06106-0 (PMC10587233; doi:10.1007/s00417-023-06106-0)
Supplement: Supplementary file 1 — Supplementary file1 (DOCX 31.1 KB) [file 417_2023_6106_MOESM1_ESM.docx]

**Diurnal functional and anatomical changes in X-linked retinoschisis**

Luca Mautone ^1^*, Yevgeniya Atiskova^1^, Druchkiv Vasyl^1^, Martin Stephan Spitzer^1^, Simon Dulz^1^

1. Department of Ophthalmology, University Medical Center Hamburg-Eppendorf, Hamburg, Germany

**Supplementary information**

***Corresponding author:**

Department of Ophthalmology

University Medical Center Hamburg-Eppendorf

Martinist. 52

20246 Hamburg, Germany

Email: l.mautone@uke.de

ORCID: 0000-0001-8323-7107

**Statistical methods of analysis**

The changes of the variables were tested either with paired T-Test or Wilcoxon Signed Rank Test depending on whether the assumptions of parametric test were met. In each case the assumptions of normality of differences were tested with Shapiro-Wilk Test. The outliers were checked using Box-Plot method and finally the assumption of homogeneous variances was checked using Levene Test. Because the changes are nested within patients, we applied mixed regression model to estimate the intrapatient correlation. This correlation was found insignificant, and so we proceeded using classical test methods including both eyes in the study. To check correlation between different parameters we applied Spearman-Rank correlation and loess smoothing to detect possible nonlinear dependencies. When comparing differences between independent groups, we applied independent T-Test or Mann-Whitney Test. All analyses were performed using R Core Team. (R Core Team 2021. R: A Language and Environment for Statistical Computing. Vienna, Austria: R Foundation for Statistical Computing. https://www.R-project.org).

**Tables**

|  | **CRT (µm)** | **Δ CRT (µm)** | **BCVA (letters)** | **Δ BCVA (letters)** | **Average threshold (dB)** | **Δ Average threshold (dB)** | **Fix stab P1 (%)** | **Fix stab P2 (%)** | **PROS length (µm)** | **Age (years)** |
| --- | --- | --- | --- | --- | --- | --- | --- | --- | --- | --- |
| **CRT (µm)** | 1.0 |  |  |  |  |  |  |  |  |  |
| **Δ CRT (µm)** | **- 0.83***** | 1.0 |  |  |  |  |  |  |  |  |
| **BCVA (letters)** | 0 | - 0.19 | 1.0 |  |  |  |  |  |  |  |
| **Δ BCVA (letters)** | 0.1 | - 0.08 | - 0.43 | 1.0 |  |  |  |  |  |  |
| **Average threshold (%)** | 0.11 | - 0.18 | **0.63*** | - 0.15 | 1.0 |  |  |  |  |  |
| **Δ Average threshold** | - 0.25 | 0.35 | - 0.37 | 0.11 | - 0.13 | 1.0 |  |  |  |  |
| **Fix stab P1 (%)** | 0.47 | - 0.06 | 0.5 | - 0.02 | 0.54 | 0.11 | 1.0 |  |  |  |
| **Fix stab P2 (%)** | 0.46 | - 0.07 | 0.48 | - 0.01 | **0.64*** | 0.18 | **0.95****** | 1.0 |  |  |
| **PROS length (µm)** | - 0.26 | 0.15 | **0.69**** | - 0.38 | **0.69**** | - 0.16 | - 0.02 | - 0.16 | 1.0 |  |
| **Age (years)** | - 0.33 | 0.12 | 0.02 | 0.18 | - 0.25 | - 0.39 | - 0.17 | - 0.29 | - 0.03 | 1.0 |

**Table S1. Correlation between morphological and functional parameters at 9 a.m. and their diurnal variation.**

Δ Average threshold: Diurnal variation of the average threshold. Δ BCVA: Diurnal variation of the Best Correlated Visual Acuity. Δ CRT: Diurnal variation of the Central Retinal Thickness. BCVA: Best Correlated Visual Acuity. CRT: Central Retinal Thickness. Fix stab: Fixation stability. PROS: Outer Photoreceptor Segment.

Spearman's rank correlation: *****p<0.0001;***p<0.001;**p<0.01;*p<0.05.*

|  | **EZ defect (N=6)** | **EZ intact (N=8)** | **Total (N=14)** | **P-Value** |
| --- | --- | --- | --- | --- |
| Δ **CRT (µm)** |  |  |  | =.**050**^b^ |
| Range | -76.00 , -8.00 | -53.00 , 31.00 | -76.00 , 31.00 |  |
| Mean (SD) | -41.00 (22.73) | -12.00 (27.00) | -24.43 (28.52) |  |
| Median (Q1,Q3) | -43.50 (-47.50, -30.50) | -12.50 (-26.25, 3.00) | -24.50 (-44.75, -8.25) |  |
| Δ **BCVA (letters)** |  |  |  | =.948^a^ |
| Range | 0.00 , 5.00 | -3.00 , 17.00 | -3.00 , 17.00 |  |
| Mean (SD) | 2.67 (1.86) | 3.62 (6.00) | 3.21 (4.58) |  |
| Median (Q1,Q3) | 3.00 (1.50, 3.75) | 3.00 (0.00, 4.25) | 3.00 (0.25, 4.00) |  |
| Δ **BCVA (LogMAR)** |  |  |  | =.289^b^ |
| Range | -0.10 , 0.50 | -0.80 , 0.50 | -0.80 , 0.50 |  |
| Mean (SD) | 0.12 (0.24) | -0.06 (0.36) | 0.01 (0.32) |  |
| Median (Q1,Q3) | 0.05 (-0.07, 0.25) | 0.00 (-0.13, 0.02) | 0.00 (-0.10, 0.10) |  |
| Δ **Average threshold (dB)** |  |  |  | =.173^b^ |
| Range | 0.60 , 5.00 | -1.20 , 2.60 | -1.20 , 5.00 |  |
| Mean (SD) | 2.60 (1.93) | 1.19 (1.44) | 1.84 (1.77) |  |
| Median (Q1,Q3) | 2.20 (1.02, 4.28) | 1.00 (0.45, 2.50) | 1.70 (0.70, 2.60) |  |

**Table S2. Correlation between integrity of the ellipsoid zone and diurnal variation of central retinal thickness, best correlated visual acuity and average threshold.**

Δ Average threshold: Diurnal variation of the average threshold. Δ BCVA: Diurnal variation of the Best Correlated Visual Acuity. Δ CRT: Diurnal variation of the Central Retinal Thickness. EZ: Ellipsoid Zone. a Mann-Whitney Test; b Independent T-Test.

|  | **ELM defect (N=10)** | **ELM intact (N=4)** | **Total (N=14)** | **P-Value** |
| --- | --- | --- | --- | --- |
| Δ **CRT (µm)** |  |  |  | =.762^b^ |
| Range | -76.00 , 31.00 | -53.00 , 0.00 | -76.00 , 31.00 |  |
| Mean (SD) | -25.80 (31.44) | -21.00 (23.17) | -24.43 (28.52) |  |
| Median (Q1,Q3) | -33.00 (-44.75, -10.00) | -15.50 (-29.75, -6.75) | -24.50 (-44.75, -8.25) |  |
| Δ **BCVA (letters)** |  |  |  | =.131^a^ |
| Range | 0.00 , 17.00 | -3.00 , 3.00 | -3.00 , 17.00 |  |
| Mean (SD) | 4.20 (4.87) | 0.75 (2.87) | 3.21 (4.58) |  |
| Median (Q1,Q3) | 3.50 (1.50, 4.75) | 1.50 (-0.75, 3.00) | 3.00 (0.25, 4.00) |  |
| Δ **BCVA (LogMAR)** |  |  |  | =.198^a^ |
| Range | -0.10 , 0.50 | -0.80 , 0.10 | -0.80 , 0.50 |  |
| Mean (SD) | 0.11 (0.24) | -0.22 (0.40) | 0.01 (0.32) |  |
| Median (Q1,Q3) | 0.00 (-0.07, 0.25) | -0.10 (-0.35, 0.02) | 0.00 (-0.10, 0.10) |  |
| Δ **Average threshold (dB)** |  |  |  | =.307^b^ |
| Range | 0.60 , 5.00 | -1.20 , 2.60 | -1.20 , 5.00 |  |
| Mean (SD) | 2.21 (1.71) | 1.00 (1.83) | 1.84 (1.77) |  |
| Median (Q1,Q3) | 1.70 (0.80, 2.70) | 1.30 (-0.15, 2.45) | 1.70 (0.70, 2.60) |  |

**Table S3. Correlation between integrity of the external limiting membrane and diurnal variation of central retinal thickness, best correlated visual acuity and average threshold.**

Δ Average threshold: Diurnal variation of the average threshold. Δ BCVA: Diurnal variation of the Best Correlated Visual Acuity. Δ CRT: Diurnal variation of the Central Retinal Thickness. ELM: External Limiting Membrane. a Mann-Whitney Test; b Independent T-Test.

|  | **COST line defect (N=2)** | **COST line intact (N=12)** | **Total (N=14)** | **P-Value** |
| --- | --- | --- | --- | --- |
| Δ **CRT (µm)** |  |  |  | =.821^b^ |
| Range | -39.00 , 0.00 | -76.00 , 31.00 | -76.00 , 31.00 |  |
| Mean (SD) | -19.50 (27.58) | -25.25 (29.78) | -24.43 (28.52) |  |
| Median (Q1,Q3) | -19.50 (-29.25, -9.75) | -24.50 (-46.50, -8.75) | -24.50 (-44.75, -8.25) |  |
| Δ **BCVA (letters)** |  |  |  | =.403^a^ |
| Range | 3.00 , 5.00 | -3.00 , 17.00 | -3.00 , 17.00 |  |
| Mean (SD) | 4.00 (1.41) | 3.08 (4.94) | 3.21 (4.58) |  |
| Median (Q1,Q3) | 4.00 (3.50, 4.50) | 3.00 (0.00, 4.00) | 3.00 (0.25, 4.00) |  |
| Δ **BCVA (LogMAR)** |  |  |  | =.079^a^ |
| Range | -0.20 , -0.10 | -0.80 , 0.50 | -0.80 , 0.50 |  |
| Mean (SD) | -0.15 (0.07) | 0.04 (0.34) | 0.01 (0.32) |  |
| Median (Q1,Q3) | -0.15 (-0.18, -0.13) | 0.00 (-0.02, 0.15) | 0.00 (-0.10, 0.10) |  |
| Δ **Average threshold (dB)** |  |  |  | NA^b^ |
| Range | 2.60 , 2.60 | -1.20 , 5.00 | -1.20 , 5.00 |  |
| Mean (SD) | 2.60 (NA) | 1.77 (1.83) | 1.84 (1.77) |  |
| Median (Q1,Q3) | 2.60 (2.60, 2.60) | 1.35 (0.68, 2.62) | 1.70 (0.70, 2.60) |  |

**Table S4. Correlation between integrity of the COST line and diurnal variation of central retinal thickness, best correlated visual acuity and average threshold.**

Δ Average threshold: Diurnal variation of the average threshold. Δ BCVA: Diurnal variation of the Best Correlated Visual Acuity. Δ CRT: Diurnal variation of the Central Retinal Thickness. COST: Cone Outer Segment Tips. a Mann-Whitney Test; b Independent T-Test.
